# Unveiling the Impact of Moderate and Severe Atopic Dermatitis: Insights on Burden, Clinical Characteristics, and Healthcare Resource Utilization in Adult Greek Patients from the APOLO Cross-Sectional Study

**DOI:** 10.3390/jcm13216327

**Published:** 2024-10-23

**Authors:** Alexander J. Stratigos, Vasiliki Chasapi, Alexander Katoulis, Efstratios Vakirlis, Fotios Psarros, Sophia Georgiou, Dimitrios Vourdas, Michael Makris, Elizabeth Lazaridou, Stamatios Gregoriou, Ioannis Skiadas, Magda Nakou, Christopher Koulias, APOLO Study Group

**Affiliations:** 1 1st Department of Dermatology and Venereology, Medical School, National and Kapodistrian University of Athens, 16121 Athens, Greece; 2 Department of Dermatology and Venereology NHS, Andreas Sygros Hospital, 16121 Athens, Greece; 3 2nd Department of Dermatology and Venereology, Medical School, National and Kapodistrian University of Athens, Attikon General University Hospital, 12462 Athens, Greece; 4 1st Department of Dermatology and Venereology, Aristotle University of Thessaloniki, 54643 Thessaloniki, Greece; 5 Department of Allergy, Athens Naval Hospital, 11521 Athens, Greece; 6 Department of Dermatology, Medical School, University of Patras, 26504 Patras, Greece; 7 Department of Allergy, 251 General Air Force Hospital, 11525 Athens, Greece; 8 Allergy Unit, 2nd Department of Dermatology and Venereology, Medical School, National and Kapodistrian University of Athens, Attikon General University Hospital, 12462 Athens, Greece; 9 2nd Department of Dermatology and Venereology, General Hospital “Papageorgiou”, Medical School Aristotle University of Thessaloniki, 56403 Thessaloniki, Greece; 10 Pfizer Hellas SA, 15451 Neo Psichiko, Greece

**Keywords:** atopic dermatitis, cross-sectional, DLQI, EASI, HCRU, work impairment

## Abstract

**Background**: Moderate to severe (M2S) atopic dermatitis (AD) is a chronic condition impacting individuals, society, and healthcare systems. Considering the changing M2S-AD treatment landscape, this study assesses the M2S-AD burden in patients reaching referral centers in Greece. **Methods**: This was a multicenter, cross-sectional study. Patients aged 12 years or older with clinically diagnosed M2S-AD were enrolled. Data collected included clinical practice assessments and the following validated patient-reported instruments: Dermatology Life Quality Index (DLQI); EuroQol-5 Dimensions-3 Level scale (EQ-5D-3L); Patient Oriented Eczema Measure (POEM); Peak Pruritus Numerical Rating Scale (PP-NRS); and Work Productivity and Activity Impairment: General Health (WPAI:GH). A pain frequency/intensity/cause questionnaire and a sleep disturbance scale were also used. **Results**: Outcomes of 184 adults (51.1% female) with M2S-AD based on the Eczema Area and Severity Index (EASI) are presented (n = 117 moderate; n = 67 severe). Among the patients, 14.8% were obese, 59.2% had allergic comorbidities, and 88.0% were receiving AD-specific therapy (systemic: 38.6%). The median age, disease duration, body surface area, and total EASI scores were 38.8 years, 11.8 years, 30.0%, and 16.9, respectively. The median DLQI score was 12.0, with ‘symptoms/feelings’ being the most affected domain. EQ-5D dimensions ‘anxiety/depression’ and ‘pain/discomfort’ were also affected (65.2% and 64.1% reporting problems, respectively). The median POEM score was 17.0. Pain, severe pruritus (PP-NRS ≥ 7), and sleep disturbance were reported by 80.4%, 62.0%, and 88.5%, respectively. The median WPAI:GH ‘work productivity loss’ and ‘activity impairment’ scores were 23.8% and 30.0%, respectively. **Conclusions:** Both moderate and severe AD patients reaching Greek specialized centers experience significant symptom burden and impairments in quality of life, sleep, work, and daily activities.

## 1. Introduction

Atopic dermatitis (AD) is a common chronic inflammatory skin disease associated with a significant patient and social burden [1,2]. Although it was previously considered a predominantly pediatric condition, recent epidemiological studies have demonstrated that atopic dermatitis is both common and burdensome among adults as well [1,2,3,4]. One-year prevalence estimates in adults range between 1.2 and 8.7% in Europe [3] and between 1.7 and 6.4% in Greece [4].

The etiopathogenesis of AD is multifactorial, involving genetic, immunologic, and environmental factors [5,6,7]. Furthermore, it is associated with both atopic (e.g., asthma, allergic rhinitis, and food allergy) and diverse non-atopic comorbidities, including infections, depression, and anxiety [1,2,8,9,10,11].

AD is characterized by dry skin, erythema, induration, lichenification, and excoriations, while patients suffer from intense pruritus and skin pain, which is an increasingly recognized symptom [1,12,13,14]. Patients also experience sleep disturbance and significant effects on their quality of life (QoL), which worsens with increasing disease severity [1,11,15,16,17,18,19,20,21,22,23,24,25,26,27,28]. Moderate-to-severe (M2S) AD poses a substantial socioeconomic burden driven by work-related challenges and increased healthcare resource utilization (HCRU) [2,16,17,18,19,29,30,31,32].

Guideline-recommended pharmacologic treatments for M2S-AD include basic emollient therapy, topical corticosteroids (TCSs), topical calcineurin inhibitors (TCIs), and systemic treatment (ST) [33,34]; current European Medicines Agency-approved systemic agents include cyclosporin, glucocorticoids, biologic agents, and Janus kinase inhibitors (JAKis), which constitute a novel addition to the therapeutic armamentarium [34,35]. Interestingly, the definition of candidates for systemic treatment has expanded and includes patients unable to participate in normal daily life activities whilst following an adequate treatment regimen, as per the social definition for candidates for systemic treatment, accentuating the patient experience and burden [34]. It is also highlighted that assessing only the signs of the disease is not an adequate tool for optimal treatment choices.

Given this changing treatment landscape of M2S-AD [36], it is crucial to understand the complex disease burden. Limited data exist for Greek patients, and the majority have been sourced from patients with self-reported disease [4,17,37]. This study aims to be the first to detail patient and disease characteristics, patient-perceived burden, and treatment patterns of M2S-AD patients following confirmed clinical diagnosis in expert centers in Greece and associated HCRU. Moreover, the results are stratified by disease severity (moderate and severe AD), allowing for further understanding of the multifaceted impact of AD on these subpopulations and the most suitable treatment selection, as per the approach described in recent guidelines.

## 2. Materials and Methods

APOLO was a multicenter, observational, cross-sectional, single-visit, primary data collection study. Eligible patients were aged ≥12 years with a confirmed diagnosis of M2S-AD as per the validated Investigator’s Global Assessment scale (vIGA-AD ≥ 3) [38], visiting the reference center either for the first time or after not attending regular follow-up for ≥2 years. The exclusion criteria included current participation in an interventional trial or the presence of other active dermatological conditions which might confound diagnosis or symptom scores. Patient and/or parental informed consent/assent were obtained, as applicable. Patients were selected consecutively.

Data were collected as part of common clinical practice, while patient-reported outcomes (PROs) were completed by patients at the start of the visit following assessment of eligibility and before any further clinical assessment(s).

The primary study objective was to assess the burden of M2S-AD using PROs as applicable to adults for AD-associated QoL via the Dermatology Life Quality Index (DLQI) [39,40,41], general health perception via the EuroQol-5 Dimensions-3 Level scale (EQ-5D-3L) [42,43], eczema severity via the Patient-Oriented Eczema Measure (POEM) [44,45,46], itching severity via the Peak Pruritus Numerical Rating Scale (PP-NRS)* [47], Work Productivity and Activity Impairment: General Health questionnaire (WPAI:GH) [48,49], and sleep disturbance [visual analogue scale (VAS) rating of average sleeplessness the previous 3 nights, ranging from 0 “No sleeplessness” to 10 “worst imaginable sleeplessness”] [50], as well as pain attributes (weekly frequency, worst intensity in the past week, and causes of pain) [24]. Secondary objectives included the description of patient and disease characteristics, treatment patterns, and AD-related HCRU. Associations of disease severity, itch intensity, sleep disturbance, and pain intensity with QoL (DLQI) were investigated as exploratory objectives. Disease severity was stratified according to the Eczema Area and Severity Index (EASI) [13].

This study followed the Guidelines for Good Pharmacoepidemiology Practice (GPP) [51], the Strengthening of the Reporting of Observational Studies in Epidemiology statement (STROBE) [52], and the local rules and regulations.

Sample size estimation was based on the precision of estimates. A sample of 200 patients was considered sufficient for the estimation of qualitative variables at a frequency of 50% where the margin of error is largest, with a half-width of 95% confidence interval <7% using the normal approximation method.

The normality of continuous variables was examined using the Shapiro–Wilk test. For uniformity purposes, the median (interquartile range; IQR) is the main summary statistic presented for non-normally distributed variables in any of the study (sub)populations. Comparison of continuous variables between two independent groups was performed using the two-sample t-test or the Mann–Whitney U-test. Associations between categorical variables were examined through the chi-squared test. The Kruskal–Wallis test was used to assess the differences in continuous/ordinal variables between the DLQI score categories. The Spearman correlation coefficient (rho) was used to assess the correlation between PROs and DLQI score. The association between DLQI and PP-NRS, sleep disturbance, total EASI, and pain intensity scores was examined with univariate and multivariate linear regression analysis. All statistical tests were two-sided at a 0.05 significance level. Statistical analysis was performed using SAS^®^ Version 9.4 (SAS Institute Inc., Cary, NC, USA).

## 3. Results

### 3.1. Patient Disposition

A total of 200 eligible patients with vIGA-based M2S-AD, including 187 adults, were consecutively enrolled by 13 referral clinics between 12 October 2021 and 30 June 2022. This study included the subsets of moderate (m-AD, n = 117) and severe/very severe (s-AD, n = 67) AD patients as per EASI, excluding three cases with mild AD as per EASI (Figure S1).

### 3.2. Patient and Disease Characteristics

In the overall M2S-AD population, the median AD-affected body surface area was 30.0% (22.0% in m-AD and 40.0% in s-AD patients), and the median EASI score was 16.9 (12.6 in m-AD and 26.0 in s-AD) (Table 1). Approximately half of the patients included reported adult-onset AD. Interestingly, both the mAD and sAD subgroups reported a median of three flare per year (defined as aggravation of symptoms requiring additional treatment), and one out of six reported more than ten flare-ups per year. Detailed patient and disease characteristics for m-AD, s-AD, and M2S-AD, as defined by the EASI scale, are summarized in Table 1, including predefined physician-diagnosed comorbidities of interest as reported by the patients. The majority of patients suffering both from m-AD and s-AD reported at least one allergic comorbidity, the most common being allergic rhinitis. Notably, one out of five and one out of three patients with m-AD and s-AD, respectively, reported suffering from clinician-diagnosed depression or anxiety disorder.

### 3.3. Dermatology-Specific Burden

**Figure 1:**
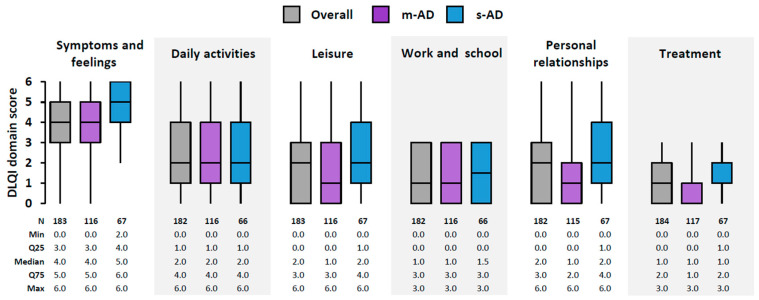
Dermatology-specific quality of life in the overall M2S-AD population and by EASI-based AD severity: DLQI domain scores. Box-plots depict median with IQR (Q25–Q75), including whiskers that extend from minimum to maximum values. AD, atopic dermatitis; DLQI, Dermatology Life Quality Index; EASI, Eczema Area and Severity Index; IQR, interquartile range; M2S, moderate-to-severe; m-AD, moderate AD; Max, maximum; Min, minimum; N, number of patients with available data; Q25, 25th percentile; Q75, 75th percentile; s-AD, severe/very severe AD.

The overall median DLQI score was 12.0 (IQR: 7.0–17.0; mean: 12.6), with 81.0% (149/184) of the patients reporting at least a moderate effect of AD (score of ≥6) and 60.9% (112/184) reporting a very large to extremely large effect (score of ≥11). The effect on QoL was significantly higher in s-AD patients compared with m-AD patients (*p* < 0.001), with a median DLQI score of 14.0 (IQR: 12.0–19.0) versus 10.0 (IQR: 5.0–16.0) and a score of ≥11 reported in 83.6% (56/67) versus 47.9% (56/117), respectively. The most affected DLQI domain was ‘symptoms and feelings’, followed by ‘daily activities’ (Figure 1).

**Figure 2:**
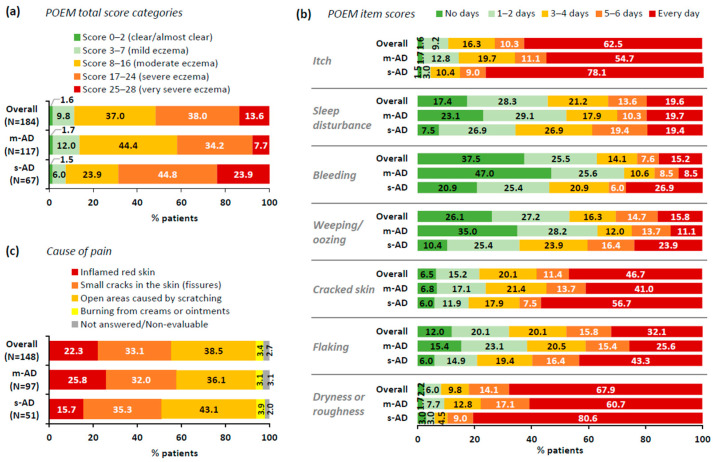
PROs on AD-related symptoms and causes of pain in the overall M2S-AD population and by EASI-based AD severity: (**a**) distribution of patients by POEM total score; (**b**) distribution of patients by POEM item score; (**c**) cause of pain among patients experiencing pain. Numbers inside bars indicate percentage (%). AD, atopic dermatitis; EASI, Eczema Area and Severity Index; M2S, moderate-to-severe; m-AD, moderate AD; N, number of patients with available data; POEM, Patient-Oriented Eczema Measure; PRO, patient-reported outcome; s-AD, severe/very severe AD; SD, standard deviation.

The overall median POEM score was 17.0 (IQR: 12.0–21.0; mean: 16.7). Median POEM scores in the m-AD and s-AD subpopulations were 16.0 (IQR: 11.0–20.0) and 20.0 (IQR: 15.0–24.0), with 41.9% and 68.7% reporting severe eczema or very severe eczema, respectively (Figure 2a). Across all POEM items, the proportion of patients experiencing eczema symptoms for ≥3 days in the last week (score ≥ 2) was higher among s-AD patients (Figure 2b). In both the m-AD and s-AD subgroups, the symptom experienced most frequently over the last week was ‘dryness or roughness’, followed by ‘itch’ and ‘cracked skin’. Notably, about 20% in both groups experienced daily sleep disturbances (Figure 2b).

AD-related pain, which is not included in the symptoms explored by the POEM, was explored independently. It was reported by 80.4% (148/184) of the overall population, with a median pain intensity of 5.0 (IQR: 3.0–7.0; mean: 5.1) on a scale of up to 10. Although the proportion of patients experiencing AD-related pain was slightly higher in m-AD patients (82.9%; 97/117) versus s-AD patients (76.1%; 51/67), pain frequency and intensity were worse among s-AD patients. Specifically, among m-AD and s-AD patients experiencing pain, 25.8% (25/97) and 37.3% (19/51) experienced pain every day, while the median pain intensity was 4.0 (IQR: 3.0–7.0) and 6.0 (IQR: 4.0–8.0), with 29.9% (29/97) and 49.0% (25/51) experiencing severe pain (intensity score ≥ 7), respectively. Causes of pain are summarized in Figure 2c.

Regarding pruritus assessed through PP-NRS, the median PP-NRS score was 7.0 (IQR: 5.0–8.0; mean: 6.7), with higher PP-NRS observed among s-AD patients (median: 8.0; IQR: 7.0–9.0) than m-AD patients (median: 7.0; IQR: 4.0–8.0). Only two patients (one m-AD, one s-AD) did not experience any itch in the previous 24 h, while 62.0% (114/184) reported severe itch (PP-NRS score ≥ 7), including 51.3% (60/117) of m-AD and 80.6% (54/67) of s-AD patients.

Based on sleep disturbance VAS rating, the majority (88.5%; 161/182) of patients reported disturbances in the last three nights (VAS score > 0), with a median VAS score of 5.0 (IQR: 2.0–7.0; mean: 5.0), corroborating POEM results. Among m-AD and s-AD patients, 84.3% (97/115) and 95.5% (64/67) reported sleep disturbances, with a median VAS score of 5.0 (IQR: 2.0–7.0) and 5.3 (IQR: 2.6–8.0), respectively.

**Figure 3:**
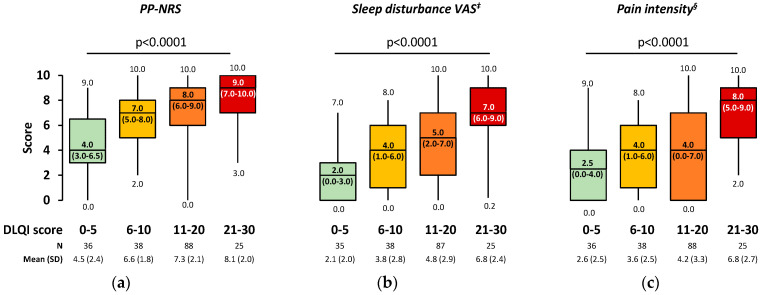
Patient-reported (**a**) PP-NRS, (**b**) sleep disturbance, and (**c**) pain intensity per DLQI score category in the overall M2S † population. Box-plots depict median with IQR (Q25–Q75), including whiskers that extend from minimum to maximum values. Numbers inside boxes indicate median (IQR). The association between categorical variables was examined through Kruskal–Wallis test. † Including 3 EASI-based mild cases. ‡ Among patients reporting sleep disturbance (VAS score > 0). § Among patients experiencing pain (at any frequency). AD, atopic dermatitis; DLQI, Dermatology Life Quality Index; EASI, Eczema Area and Severity Index; IQR, interquartile range; N, number of patients with available data; PP-NRS, Peak Pruritus Numerical Rating Scale; Q25, 25th percentile; Q75, 75th percentile; SD, standard deviation; VAS, visual analogue scale.

After adjusting for baseline factors of interest, multivariable analysis revealed that for a one-unit increase in EASI total score, the DLQI total score is expected to increase by 0.12 units on average (Table 2). A negligible positive correlation between EASI and DLQI score was demonstrated (rho = 0.251; *p* < 0.001). Additionally, multivariable analysis demonstrated that for a one-unit increase in PP-NRS, sleep disturbance VAS, and pain intensity score, the DLQI score is expected to increase by 1.11, 0.96 and 0.64 units on average, respectively (Table 2). A statistically significant low positive correlation was observed between DLQI and the aforementioned PROs, specifically PP-NRS (rho = 0.459; *p* < 0.001), sleep disturbance VAS (rho = 0.483; *p* < 0.001), and pain intensity score (rho = 0.364; *p* < 0.001). All the aforementioned PROs were statistically significantly higher with increasing DLQI score category (Figure 3).

### 3.4. Health-Related QoL (HRQoL)

The mean (SD) EQ-5D-3L utility index and EQ-VAS scores in the overall population were 0.7 (0.2) and 67.2 (21.9), respectively. The respective scores in m-AD patients were 0.7 (0.2) and 67.7 (21.9), and in s-AD patients 0.7 (0.2) and 66.3 (22.1). The most adversely affected dimensions were ‘anxiety/depression’ and ‘pain/discomfort’, with 65.2% (120/184) and 64.1% (118/184) of patients reporting problems, respectively (Figure S2).

### 3.5. Work Productivity and Activity Impairment

Among students and employed or self-employed patients with available data, 29.7% (44/148) reported missing school or work due to AD for at least 2 days in the last month; the percentages among m-AD and s-AD patients were 20.2% (19/94) and 46.3% (25/54), respectively. WPAI:GH outcomes in the overall population and among those reporting missed time or impairment are presented in Table 3. Interestingly, although absenteeism and presenteeism are more often reported in s-AD than m-AD patients, the impact on those reporting is similar in the two subgroups.

### 3.6. Treatments

**Figure 4:**
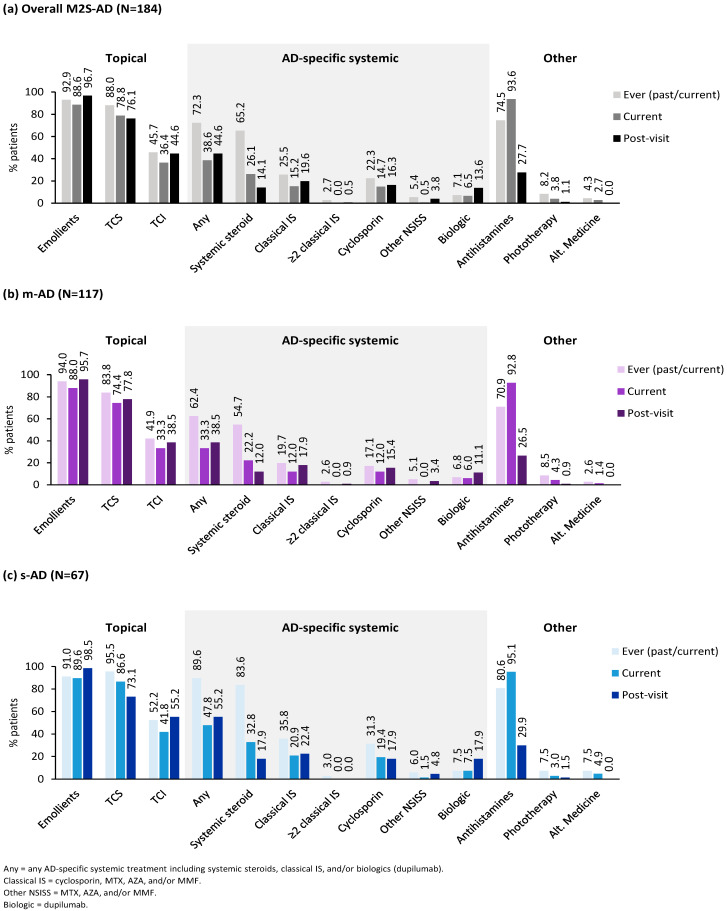
Treatments ever used, treatments used 2 weeks prior to expert center visit (current), and treatments selected by expert centers (post-visit), (**a**) in the overall M2S-AD population, (**b**) in the EASI-based moderate AD subpopulation, and (**c**) in the EASI-based severe/very severe AD subpopulation. AD, atopic dermatitis; Alt, alternative; AZA, azathioprine; EASI, Eczema Area and Severity Index; IS, immunosuppressants; M2S, moderate-to-severe; m-AD, moderate AD; MMF, mycophenolate mofetil; MTX, methotrexate; NSISS, non-steroidal immunosuppressants; s-AD, severe/very severe AD; TCI, topical calcineurin inhibitor; TCS, topical corticosteroid.

Treatments ever used, currently used (within the last 2 weeks), and suggested post-visit are summarized in Figure 4. Examining the systemic corticosteroid use in the last year prior to the visit, 36.5% (65/178) of the overall M2S-AD patients received at least two courses of systemic steroids. Specifically, 28.3% (32/113) of m-AD and 50.8% (33/65) of s-AD patients received ≥2 systemic steroid courses in the last year prior to the visit. Additional details of past treatments and patients’ out-of-pocket expenses for the management of their disease are provided in Table S1.

At the time of the visit, 82.1% of patients reported the use of at least one topical treatment (77.8% and 89.5% of m-AD and s-AD patients, respectively). Proactive use of topical corticosteroids (TCSs) was reported by 20.7% of the patients (18.8% of m-AD and 23.9% of s-AD patients). Proactive use was also noted for topical calcineurin inhibitors (TCIs) at a rate of 15.2%, which was more frequent in s-AD than m-AD patients (22.4% vs. 11.1%). Moreover, most M2S AD patients (51.3% in m-AD and 56.7% in s-AD) required special topical treatment regimens in specific areas of the body which were different to the topical treatments used in other body areas involved, adding to the complexity of topical treatments required. The proportion of patients receiving AD-specific systemic treatment (ST), including systemic steroids, within 2 weeks prior to the visit was 38.6%. Rates of recent AD-specific ST were higher among s-AD than m-AD patients (Figure 4).

Post-visit, 84.2% of patients were prescribed ≥1 topical treatment, with proactive TCS and TCI rates of 32.1% and 22.3%. In more detail, of the m-AD and s-AD patients, 82.9% and 86.6% were offered topical treatment post-visit, respectively, with proactive TCS rates of 28.2% in m-AD and 38.8% in s-AD patients and proactive TCI rates of 14.5% in m-AD and 35.8% in s-AD patients. Additionally, the percentage of patients receiving a different topical treatment for specific areas of the body slightly increased to 58.7% (58.1% for m-AD and 59.7% for s-AD), compared to initial regimens used. Additionally, the percentage of AD-specific ST increased to 44.6%, including a slight increase in the use of classical immunosuppressants and doubling the use of biologics in the overall population. Systemic steroids were also offered to 14.1% of patients. The relevant percentages of m-AD and s-AD patients are included in Figure 4. Of note, the use of antihistamines significantly dropped post-visit to expert centers.

### 3.7. AD-Related HCRU

The most common diagnosing physician specialty was the ‘dermatologist’, reported in 71.7% (132/184) of patients, followed by the ‘allergist’ in 14.1% (26/184), ‘pediatrician’ in 13.0% (24/184), and ‘general practitioner’ in two patients (1.1%). Over the past year prior to expert center consultation, 95.7% (176/184) of patients had consulted a median of 2.0 (1.0–2.0) different HCP specialties (including pharmacists) and performed a median (IQR) of 5.0 (3.0–10.0) visits [4.0 (2.0–6.0) visits excluding pharmacists]. The most common physician specialties were dermatologists [median (IQR) visits: 2.0 (2.0–4.0) for the overall and m-AD and s-AD subpopulations] and allergists [median (IQR) visits: overall 2.0 (1.0–4.0); m-AD 2.0 (1.0–4.0); s-AD 2.5 (1.0–8.5)] (Figure S3).

Over the past year prior to expert center consultation, 62.1% (113/182), 28.0% (51/182), and 6.6% (12/183) of evaluable patients had performed a median of 3.0 (IQR: 1.0–4.0) AD-related hospital/specialized center consultations (total of 358 consultations), 2.0 (IQR: 1.0–2.0) AD-related emergency room (ER) visits (total of 108 visits), and 1.0 (IQR: 1.0–1.0) AD-related hospitalization (total of 14 hospitalizations), respectively. The overall frequency of AD-related ER visits and hospitalizations in the past (ever performed) was 36.5% (66/181; total ER visits: 283; median: 3.0; IQR: 1.0–5.0) and 12.6% (23/182; total hospitalizations: 39; median: 1.0; IQR: 1.0–1.0), respectively.

The rate of AD-related hospital/specialized center consultations over the past year was similar between m-AD (61.2%; 71/116; total consultations: 226) and s-AD patients (63.6%; 42/66; total consultations: 132). Conversely, a higher frequency of ER visits and hospitalizations due to AD was observed among s-AD patients than m-AD patients. Specifically, among m-AD and s-AD patients, 22.4% (26/116) and 37.9% (25/66) reported any AD-related ER visits in the last year (total of 53 and 55 visits, respectively), while five (4.3%) and seven (10.4%) patients reported any AD-related hospitalization in the last year (total of seven hospitalizations in both subpopulations), respectively. The overall frequency of AD-related ER visits and hospitalizations in the past (ever performed) among m-AD patients was 28.7% (33/115) and 11.2% (13/116), respectively, while the respective proportions for s-AD patients were 50.0% (33/66) and 15.2% (10/66).

**Figure 5:**
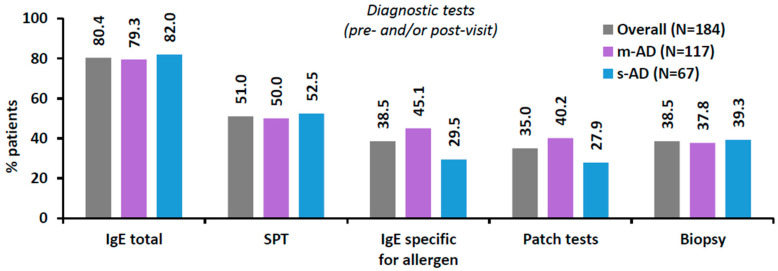
Diagnostic tests performed for AD in the past and/or ordered by the specialized centers at the study visit in the overall M2S-AD population and by EASI-based AD severity. Numbers on top of the bars indicate percentage (%). AD, atopic dermatitis; EASI, Eczema Area and Severity Index; M2S, moderate-to-severe; m-AD, moderate AD; N, number of patients with available data; s-AD, severe/very severe AD.

Of the overall population, 54.3% (100/184) were referred to the study site by a healthcare professional (HCP); most commonly by a dermatologist (77.0%), a general practitioner (8.0%), or an allergist (7.0%). The frequencies of required patch testing, biopsies and other AD-related diagnostic tests (pre- and/or post-visit) are presented in Figure 5. Of note, almost 4 out of 10 M2S-AD patients required biopsy at some point.

## 4. Discussion

The APOLO study generated novel real-world (RW) evidence on the burden, treatments patterns, and patient journey of physician-diagnosed M2S-AD.

Our results confirm the high burden of both moderate and severe AD on dermato-logy-specific and generic QoL. AD had at least a moderate effect on QoL based on DLQI in 81% of patients, similar to the 72–84% reported by Gregoriou et al. (2022; referred hereinafter as the Greek study), which reported a similar mean DLQI score (10.1) [17]. This rate was higher than the proportion reported for the EU5 study pooled data (40%) [53]. Notably, the effect of AD was very large in more than 80% of s-AD patients in APOLO. The DLQI scores of m-AD and s-AD were consistent with other European countries (mean: 6.0–10.0 [15,18] and 12.0–18.9 [18,28] respectively). In APOLO, EASI and DLQI scores were weakly correlated (rho: 0.25), similarly to the pooled baseline results of dupilumab phase 3 trials among M2S-AD patients (rho: 0.29) [54]. However, regression analysis adjusted for baseline factors demonstrated that increased AD severity is associated with a declining QoL, like in other RW studies [15,17,55].

Patients with AD suffer from eczema, the presence of which has been associated with an increased risk of depression and anxiety [56], both impacting an individual’s HRQoL. The EQ-5D-3L utility index and EQ-VAS findings in APOLO indicate that M2S-AD patients have a lower HRQoL compared with the general Greek population (scores of 0.81 and 74.7, respectively) [43]. A significant proportion of M2S-AD patients reported anxiety/depression (65%), and pain/discomfort (64%), which were equally high in both m-AD and s-AD subpopulations, unlike the respective general Greek norms of 45% and 28% [43].

Regarding the signs and symptoms of AD, a combination of which is necessary for AD severity characterization, multiple outcome measures were utilized in APOLO. In this study, POEM-based moderate eczema severity was more frequently reported than previously reported for M2S-AD in other European countries (~70%) [28]. Consistently, POEM scores were higher than those reported in two M2S-AD RW studies (mean: 10.6–13.1 for overall M2S-AD [18]; 8.8–12.7 for m-AD [15,18]; 12.2–18.4 for s-AD [18]). APOLO’s results indicated that pain intensity was similar to baseline intensity in M2S-AD clinical trials (mean scores: 5.8–6.6 [26]) but higher than that of general AD (any severity) as measured in a US skin-pain NRS validation study (median score: 2.0) [57]. The majority of APOLO patients (80%) experienced pain over the past week, indicating it is a key symptom. Daily pain was reported by 30%, with similar rates between m-AD and s-AD, highlighting that even m-AD patients experience pain frequently.

Nearly two-thirds of APOLO patients experienced severe pruritus (PP-NRS ≥ 7; 62%) and daily itching (POEM score 4; 63%), including the majority of m-AD patients. Of note, 40% of m-AD patients were classified as severe (score > 16) per POEM. Furthermore, 96% of patients experienced a median of 3.0 flare-ups in the last year. The mean PP-NRS in APOLO was consistent with that of general AD (any severity) across European countries in a pruritus intensity scale validation study (mean: 6.7) [58] and with baseline levels in M2S-AD clinical trials (mean: 6.5–7.2) [26]. Notably, more APOLO patients experienced sleep disturbance compared with other European studies (54–69%) [17,19,53]. Self-reported PP-NRS, pain intensity, and sleep disturbance were positively correlated and significantly associated with DLQI in APOLO. Sleep disturbance has been previously associated with worse HRQoL (EQ-5D) [19], while a positive correlation between pain intensity and DLQI is supported by RW US research [24,25] and M2S-AD international clinical trials [26,27]. The above results support the conclusion that M2S-AD patients are burdened by severe pruritus, sleep disturbance, and pain, which affect their QoL; hence, addressing these is an important patient-reported treatment goal [59] and a persistent challenge in AD management.

Moreover, APOLO highlighted the impact on productivity. ‘Work productivity loss’ and ‘activity impairment’ scores in APOLO were worse than those in non-AD European patients (23.5 and 27.9%, respectively) [53]. APOLO scores were within the range of European RW evidence in the literature for M2S-AD (mean: 20.2–33.1% and 25.4–37.7%) [18,53] but slightly better than for M2S-AD patients in the Greek study (mean: 31.2–48.5% and 30.6–39.3%) [17]. Interestingly, emerging data suggest that pruritus severity is associated with sleep disturbance, which in turn is associated with work productivity [60].

Most patients (82%) in APOLO were receiving TCIs and/or TCSs, while a considerable proportion received AD-specific ST (39%), mainly systemic steroids and classical immunosuppressants. The absence of targeted small molecules like (JAKis) and the low utilization of biologics likely reflects the limited period of availability and reimbursement status of these agents in Greece at the time of data collection. This is also supported by the doubling in prescription rates of biologics post-study (14% versus past/current rate of 7%). These data may serve as a benchmark for the future uptake of newly approved therapies, including injectable biologics and oral JAKis, which have shown important improvements in QoL, anxiety/depression, and sleep disturbance in M2S-AD patients [27,61,62,63,64,65]. With regard to selection of systemic treatment, our study offers insights on specific comorbidities that may affect the selection of JAK inhibitors, such as history of venous thromboembolism or cancer.

Lastly, despite the growing body of evidence on HCRU in Europe [2], Greek data are scarce [4,17]. Considering the intercountry differences in healthcare accessibility, local data remain necessary to capture the impact of AD in specific healthcare settings. APOLO provides insights into the patient journey before resorting to expert centers, given that the participants were suffering from AD symptoms for a median of 12 years before the study visit. In the last year, 91% of patients had performed a mean of 6.2 AD-related HCP visits (excluding pharmacists), higher than the 2.6–2.9 range reported for the last year in the Greek study [17]. This difference may be partly attributed to the fact that the APOLO population comprised physician-diagnosed AD patients, in contrast to the self-reported AD population of the Greek study [4,17]. Although s-AD patients had higher rates of hospitalization and ER visits (10% and 38% versus 4% and 22% in m-AD), both m-AD and s-AD patients attended a similar number of HCP visits and hospital/specialized center consultations in the last year, demonstrating the high HCRU by m-AD patients as well.

Altogether, the above findings join the published literature showing a significant humanistic burden among M2S-AD patients. The comparisons described above should be cautiously interpreted. The differences in study designs (telephone or web-based surveys [15,17,19,28,53]), AD severity rating methods (Patient-Oriented SCORing Atopic Dermatitis/PO-SCORAD-based [15]; POEM-based [17]; physician-reported [18]; DLQI-based [19]; self-reported [28,53]), distribution between moderate and severe cases (m-AD proportion: 75% [15]; 78–84% [18]; 59% [19]; 79% [28]; 87% [53]; not available [17]), and healthcare settings (referral expert centers versus physician practices) should be considered.

The limitations of this study include selection bias, confounding, and information/misclassification bias. Patient selection bias was mitigated by consecutive enrollment. Information bias is expected to be minimal, considering the low missing data rate (<1%) and short recall period (≤7 days) of PROs. Nonetheless, past HCRU estimates may be affected by recall bias, while those pertaining to the last year before the visit may have been underestimated as a result of the COVID-19 pandemic-related public health/social measures taken, including transport restrictions and healthcare access constraints. Though the extent and persistence of the COVID-19 pandemic’s negative effects on the subjects’ well-being are unclear [66,67,68,69], they should be taken into consideration when interpreting this study’s HRQoL-related findings. Furthermore, in the absence of statistical comparisons, any observed similarities/differences between the m-AD and s-AD subpopulations are purely descriptive. It should be further noted that, although no specific diagnosis criteria were enforced for study participation, this is not expected to have introduced any bias since clinical diagnosis was confirmed at the expert centers. On the other hand, the fact that the APOLO study included patients seeking consultation for the first time in expert centers may partly account for the high severity scores and PROs; hence, the results might not be entirely generalizable to the M2S-AD population encountered in routine care settings. Moreover, since this study was conducted solely within Greece, the findings may not be fully generalizable to other settings. Factors such as the specific ethnic composition, healthcare access, socioeconomic background, and cultural attitudes prevalent in Greece could affect the captured burden of atopic dermatitis. Consequently, the results should be interpreted with caution when extrapolating to populations with different demographics and healthcare system profiles.

## 5. Conclusions

In conclusion, both m-AD and s-AD patients reaching specialized centers in Greece experience significant symptom burden and impairments in QoL, work productivity, daily activities, and sleep quality, albeit the vast majority are pharmacologically treated and have been on a years-long journey since initial diagnosis. These findings highlight the unmet therapeutic needs of this population.

## Figures and Tables

**Table 1:** Patient and disease characteristics in the overall M2S-AD population and by severity.

		Overall (N = 184)	m-AD (N = 117)	s-AD (N = 67)
Sociodemographic and anthropometric characteristics at the study visit *				
Age (years)	median (IQR)	38.8 (24.7–52.7)	39.2 (25.2–52.7)	38.4 (24.3–52.5)
	<65, n (%)	164 (89.1)	105 (89.7)	59 (88.1)
Female, n (%)		94 (51.1)	64 (54.7)	30 (44.8)
BMI (kg/m^2^), median (IQR) **		25.0 (22.2–27.8)	24.8 (21.5–27.9)	25.3 (22.7–27.2)
BMI classification, n (%)	Underweight (BMI < 18.5 kg/m^2^)	4 (2.2)	3 (2.6)	1 (1.5)
	Normal (18.5 ≤ BMI < 25 kg/m^2^)	87 (47.3)	57 (48.7)	30 (44.8)
	Overweight (25 ≤ BMI < 30 kg/m^2^)	65 (35.3)	38 (32.5)	27 (40.3)
	Obese (BMI ≥ 30 kg/m^2^)	27 (14.7)	18 (15.4)	9 (13.4)
	Missing/Not assessed	1 (0.5)	1 (0.9)	.
Smoking (current), n (%)		52 (28.3)	30 (25.6)	22 (32.8)
**Family history and physician-diagnosed comorbidities of interest**				
Family history of AD and/or other allergic conditions, n (%)		101 (54.9)	65 (55.6)	36 (53.7)
History of allergic comorbidity (allergic rhinitis/allergic conjunctivitis/asthma/food allergy), n (%)	Any	109 (59.2)	65 (55.6)	44 (65.7)
	Allergic rhinitis	82 (44.6)	46 (39.3)	36 (53.7)
	Allergic conjunctivitis	63 (34.2)	35 (29.9)	28 (41.8)
	Asthma	46 (25.0)	24 (20.5)	22 (32.8)
	Food allergy	27 (14.7)	15 (12.8)	12 (17.9)
Depression or anxiety disorder, n (%)		49 (26.6)	25 (21.4)	24 (35.8)
Anxiety disorder, n (%)		42 (22.8)	23 (19.7)	19 (28.4)
Contact dermatitis, n (%)		41 (22.3)	26 (22.2)	15 (22.4)
History of Herpes simplex infections, n (%)		33 (17.9)	21 (17.9)	12 (17.9)
Hypertension, n (%)		27 (14.7)	17 (14.5)	10 (14.9)
History of skin infections (requiring antibiotics), n (%)		25 (13.6)	12 (10.3)	13 (19.4)
Depression, n (%)		20 (10.9)	10 (8.5)	10 (14.9)
Diabetes, n (%)		10 (5.4)	7 (6.0)	3 (4.5)
Alopecia areata, n (%)		9 (4.9)	8 (6.8)	1 (1.5)
History of Herpes zoster infections, n (%)		8 (4.3)	4 (3.4)	4 (6.0)
Inflammatory bowel disease, n (%)		6 (3.3)	5 (4.3)	1 (1.5)
History of venous thromboembolism (VTE), n (%)		2 (1.1)	.	2 (3.0)
Cancer, n (%)		1 (0.5)	1 (0.9)	.
History of cerebrovascular accident (stroke), n (%)		1 (0.5)	1 (0.9)	.
**Disease characteristics**				
Age at onset of AD (years)	median (IQR)	19.0 (5.5–39.1)	19.7 (6.3–37.4)	16.4 (4.6–45.6)
	≥18, n (%)	97 (52.7)	65 (55.6)	32 (47.8)
Time from onset to diagnosis (months), median (IQR)		1.9 (0.0–21.2)	0.5 (0.0–12.0)	12.0 (0.0–26.3)
Disease duration (from onset to study visit) (years), median (IQR)		11.8 (4.5–25.7)	12.1 (5.1–26.1)	11.5 (4.1–22.3)
AD-affected BSA at the study visit (%), median (IQR)		30.0 (20.0–40.0)	22.0 (15.0–40.0)	40.0 (30.0–60.0)
Face/neck involvement at the study visit	face, n (%)	123 (66.8)	78 (66.7)	45 (67.2)
	neck, n (%)	91 (49.5)	52 (44.4)	39 (58.2)
Total EASI score at the study visit	mean (SD)	18.7 (10.1)	12.7 (4.2)	29.2 (8.8)
	median (IQR)	16.9 (10.4–23.4)	12.6 (8.9–16.2)	26.0 (22.8–32.4)
vIGA-AD at the study visit	3, n (%)	125 (67.9)	97 (82.9)	28 (41.8)
	4, n (%)	59 (32.1)	20 (17.1)	39 (58.2)
History of flare-ups in the last year	median (IQR)	3.0 (2.0–6.0)	3.0 (2.0–6.0)	3.0 (3.0–6.0)
	≥1 flare-up(s), n (%)	176 (95.7)	113 (96.6)	63 (94.0)
	≥3 flare-up(s), n (%)	127 (69.0)	76 (65.0)	51 (76.2)
	≥10 flare-ups, n (%)	30 (16.3)	19 (16.2)	11 (16.4)

* All patients were white (Europe, Russia, Middle East, North Africa, USA, Canada, Australia). ** BMI is missing for one patient with EASI-based moderate AD. Abbreviations: AD, atopic dermatitis; BMI, body mass index; BSA, body surface area; EASI, Eczema Area and Severity Index; IQR, interquartile range; M2S, moderate-to-severe; m-AD, moderate AD; n, number of patients with variable; N, number of patients with available data; s-AD, severe/very severe AD; SD, standard deviation; vIGA-AD, validated Investigator’s Global Assessment.

**Table 2:** Association of EASI and PROs with DLQI total score through univariate linear regression model adjusted for baseline factors of interest through multivariate linear regression model in the overall M2S population.

	OLS Estimates (of Mean DLQI Total Score)				
	n	Crude Betas (95% CI)	*p*-Value	Adjusted Betas (95% CI)	*p*-Value
EASI score	187 *	0.18 (0.09–0.27)	<0.001	0.12 (0.00–0.23) ^†^	0.044
PP-NRS total score	187 *	1.34 (0.97–1.71)	<0.001	1.11 (0.74–1.47) ^‡^	<0.001
Sleep disturbance (VAS)	185 **	1.10 (0.80–1.39)	<0.001	0.96 (0.67–1.26) ^‡^	<0.001
Pain intensity NRS score	187 *	0.87 (0.57–1.16)	<0.001	0.64 (0.34–0.94) ^‡^	<0.001

* Including 3 EASI-based mild cases. ** Including 1 EASI-based mild case. ^†^ Adjusted for baseline factors of interest: age at study visit (years; continuous), sex, educational status (≥Bachelor’s versus <Bachelor’s degree), employment status (employed versus other), BMI (kg/m^2^; continuous), duration of AD (years; continuous), BSA (%; continuous). ^‡^ Adjusted for baseline factors of interest: age at study visit (years; continuous), sex, educational status (≥Bachelor’s versus <Bachelor’s degree), employment status (employed versus other), BMI (kg/m^2^; continuous), vIGA-AD score (3 versus 4), duration of AD (years; continuous), BSA (%; continuous). Abbreviations: AD, atopic dermatitis; BSA, body surface area; CI, confidence interval; DLQI, Dermatology Life Quality Index; EASI, Eczema Area and Severity Index; M2S, moderate-to-severe; n, number of patients with variable; NRS, numerical rating scale; OLS, ordinary least squares; PP-NRS, Peak Pruritus Numerical Rating Scale; PRO, patient-reported outcome; VAS, visual analogue score; vIGA, validated Investigator’s Global Assessment.

**Table 3:** Work productivity and activity impairment in the past seven days as assessed by WPAI:GH in the overall M2S-AD population and by EASI-based AD severity.

		Overall (N = 184)	m-AD (N = 117)	s-AD (N = 67)
**All patients**				
Employed, n		112	72	40
Absenteeism	Completion rate, % (n/N)	97.3 (109/112)	95.8 (69/72)	100.0 (40/40)
	Mean (SD) score, %	4.9 (12.0)	4.9 (14.1)	4.7 (7.2)
	Median (IQR) score, %	0.0 (0.0–4.8)	0.0 (0.0–2.0)	0.0 (0.0–6.0)
Presenteeism	Completion rate, % (n/N)	99.1 (111/112)	98.6 (71/72)	100.0 (40/40)
	Mean (SD) score, %	27.1 (26.3)	25.8 (27.0)	29.5 (25.3)
	Median (IQR) score, %	20.0 (0.0–40.0)	20.0 (0.0–40.0)	20.0 (10.0–45.0)
Work productivity loss	Completion rate, % (n/N)	97.3 (109/112)	95.8 (69/72)	100.0 (40/40)
	Mean (SD) score, %	29.8 (27.3)	28.0 (28.5)	32.8 (25.2)
	Median (IQR) score, %	23.8 (2.4–50.0)	20.0 (0.0–46.6)	26.9 (10.0–51.0)
Activity impairment	Completion rate, % (n/N)	100.0 (184/184)	100.0 (117/117)	100.0 (67/67)
	Mean (SD) score, %	34.2 (30.0)	29.5 (28.6)	42.4 (30.7)
	Median (IQR) score, %	30.0 (5.0–60.0)	20.0 (0.0–50.0)	40.0 (20.0–70.0)
**Patients reporting any work productivity loss/activity impairment**				
Absenteeism	Patients reporting absenteeism, % (n/N)	34.9 (38/109)	27.5 (19/69)	47.5 (19/40)
	Mean (SD) score, %	13.9 (17.1)	17.9 (22.5)	9.9 (7.6)
	Median (IQR) score, %	7.0 (4.8–21.1)	11.1 (4.0–23.7)	6.5 (4.8–14.3)
Presenteeism	Patients reporting presenteeism, % (n/N)	71.2 (79/111)	66.2 (47/71)	80.0 (32/40)
	Mean (SD) score, %	38.1 (23.5)	38.9 (24.2)	36.9 (22.9)
	Median (IQR) score, %	30.0 (20.0–60.0)	30.0 (20.0–60.0)	30.0 (20.0–55.0)
Work productivity loss	Patients reporting work productivity loss, % (n/N)	75.2 (82/109)	69.6 (48/69)	85.0 (34/40)
	Mean (SD) score, %	39.6 (24.5)	40.3 (25.9)	38.6 (22.7)
	Median (IQR) score, %	33.3 (20.0–60.0)	34.1 (20.0–60.0)	31.7 (22.0–55.6)
Activity impairment	Patients reporting activity impairment, % (n/N)	75.0 (138/184)	70.9 (83/117)	82.1 (55/67)
	Mean (SD) score, %	45.6 (26.0)	41.6 (25.5)	51.6 (25.8)
	Median (IQR) score, %	40.0 (20.0–70.0)	40.0 (20.0–60.0)	50.0 (30.0–70.0)

Abbreviations: AD, atopic dermatitis; EASI, Eczema Area and Severity Index; IQR, interquartile range; M2S, moderate-to-severe; m-AD, moderate AD; n, number of patients with variable; N, number of patients with available data; s-AD, severe/very severe AD; SD, standard deviation; WPAI:GH, Work Productivity and Activity Impairment: General Health (questionnaire).

## Data Availability

The data that support the findings of this study are available from Pfizer Hellas, but restrictions apply to the availability of these data, which were used under license for the current study and so are not publicly available. Data are, however, available from the corresponding author upon reasonable request and with permission of Pfizer.

## Supplementary Materials

The following supporting information can be downloaded at https://www.mdpi.com/article/10.3390/jcm13216327/s1, Table S1: Treatment details and costs incurred by patients in the overall M2S-AD population and by EASI-based AD severity; Figure S1: Patient disposition; Figure S2: Proportion of patients with reported problems for each level on each dimension of the EQ-5D-3L in the overall M2S-AD population and by EASI-based AD severity; Figure S3: HCP specialties consulted in the past in the overall M2S-AD population and by EASI-based AD severity.
