# Supplementary material for: Unveiling the Impact of Moderate and Severe Atopic Dermatitis: Insights on Burden, Clinical Characteristics, and Healthcare Resource Utilization in Adult Greek Patients from the APOLO Cross-Sectional Study
Source: J Clin Med. 2024 Oct 23;13(21):6327. doi: 10.3390/jcm13216327 (PMC11546607; doi:10.3390/jcm13216327)
Supplement: Supplementary file 1 [file jcm-13-06327-s001.zip › jcm-3226972-supplementary.pdf]

Supplementary Materials

Supplementary Table S1. Treatment details and costs incurred by patients in the overall M2S-AD population and by EASI-based AD severity.

Supplementary Figure S1. Patient disposition.

Supplementary Figure S2. Proportion of patients with reported problems for each level on each dimension of the EQ-5D-3L, in the overall M2S-AD population and by EASI-based AD severity.

Supplementary Figure S3. HCP specialties consulted in the past, in the overall M2S-AD population and by EASI-based AD severity.

Supplementary Table S1. Treatment details and costs incurred by patients in the overall M2S-AD population and by EASI-based AD severity.

		Overall (N=184)	m-AD (N=117)	s-AD (N=67)
**Use of TCS/TCI in the last 6 months**				
TCS	Yes, n (%)	160 (87.0)	97 (82.9)	63 (94.0)
	Number of tubes, median (IQR)	4.0 (2.0-6.0)	2.0 (2.0-5.0)	5.5 (3.0-10.0)
TCI	Yes, n (%)	81 (44.0)	47 (40.2)	34 (50.7)
	Number of tubes, median (IQR)	2.0 (1.0-3.0)	2.0 (1.0-3.0)	2.0 (1.0-2.0)
**Systemic steroid courses in the past**				
Receipt of systemic steroids in the past (total)	Yes, n (%)	108 (58.7)	59 (50.4)	49 (73.1)
	>10 courses, n (%)	32 (18.5)	14 (12.7)	18 (28.6)
Receipt of systemic steroids in the last year	Yes, n (%)†	100 (56.2)	51 (45.1)	49 (75.4)
	Number of courses, median (IQR)	1.0 (0.0-3.0)	0.0 (0.0-2.0)	2.0 (1.0-3.0)
**Out-of-pocket cost of emollients and AD-related skin care products used within the last month**				
€, median (IQR)		30.0 (20.0-40.0)	25.0 (20.0-35.0)	30.0 (20.0-40.0)
**Cost of items purchased by the patients for better management of AD**				
≤50€, n (%)		75 (40.8)	52 (44.4)	23 (34.3)
51-100€, n (%)		65 (35.3)	42 (35.9)	23 (34.3)
101-200€, n (%)		23 (12.5%)	10 (8.5%)	13 (19.4%)
>200€, n (%)		21 (11.4%)	13 (11.1%)	8 (11.9%)
**Special location (sensitive area) requiring different post-visit topical treatment than other body areas involved**				
Yes, n (%)		108 (58.7%)	68 (58.1%)	40 (59.7%)

†Excluding 6 patients (4 with moderate and 2 with severe/very severe disease) with missing data.
AD, atopic dermatitis; EASI, eczema area and severity index; IQR, interquartile range; M2S, moderate-to-severe; m-AD, moderate AD; n, number of patients with variable; N, number of patients with available data; s-AD, severe/very severe AD; SD, standard deviation; TCI, topical calcineurin inhibitor; TCS, topical corticosteroid.

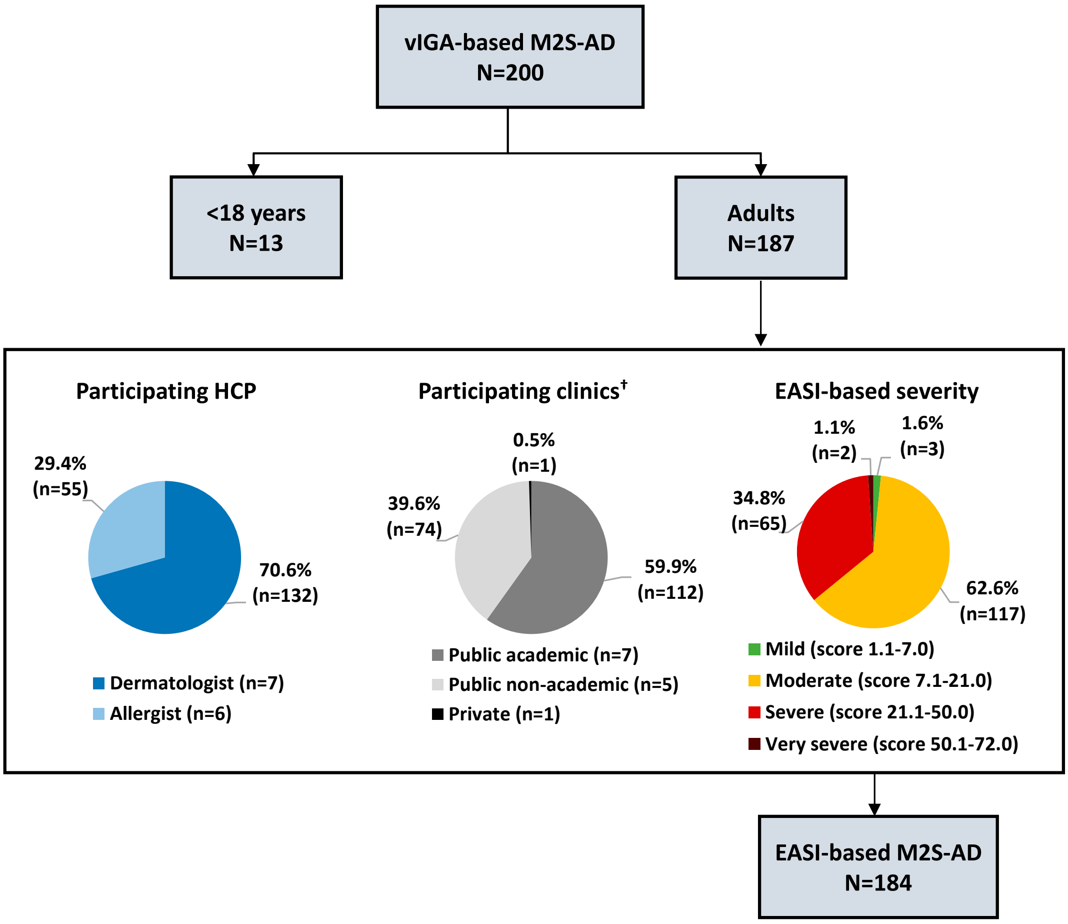

Supplementary Figure S1. Patient disposition.

†Total of 11 hospital institutions.

AD, atopic dermatitis; EASI, eczema area and severity index; HCP, healthcare professional; M2S, moderate-to-severe; vIGA, validated investigator's global assessment.

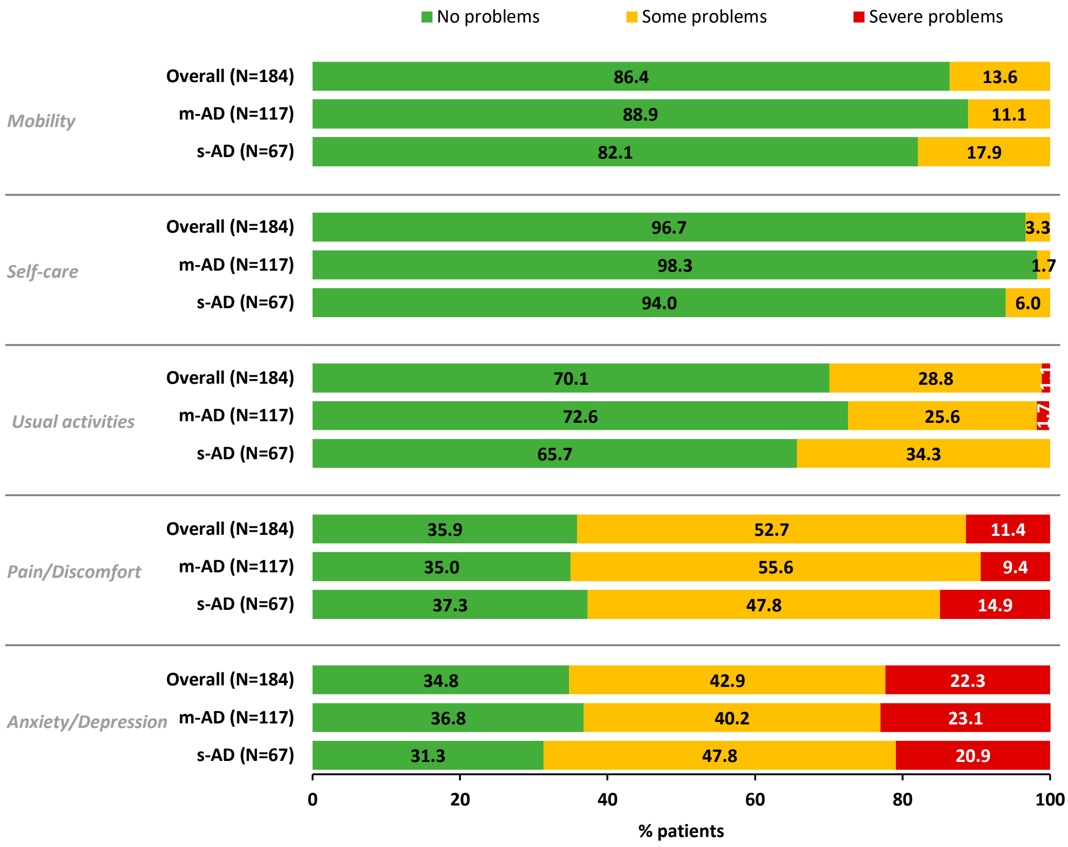

Supplementary Figure S2. Proportion of patients with reported problems for each level on each dimension of the EQ-5D-3L, in the overall M2S-AD population and by EASI-based AD severity.

Numbers inside bars indicate percentage (%).

AD, atopic dermatitis; EASI, eczema area and severity index; EQ-5D-3L, EuroQol-5 dimensions-3 levels; M2S, moderate-to-severe; m-AD, moderate AD; N, number of patients with available data; s-AD, severe/very severe AD.

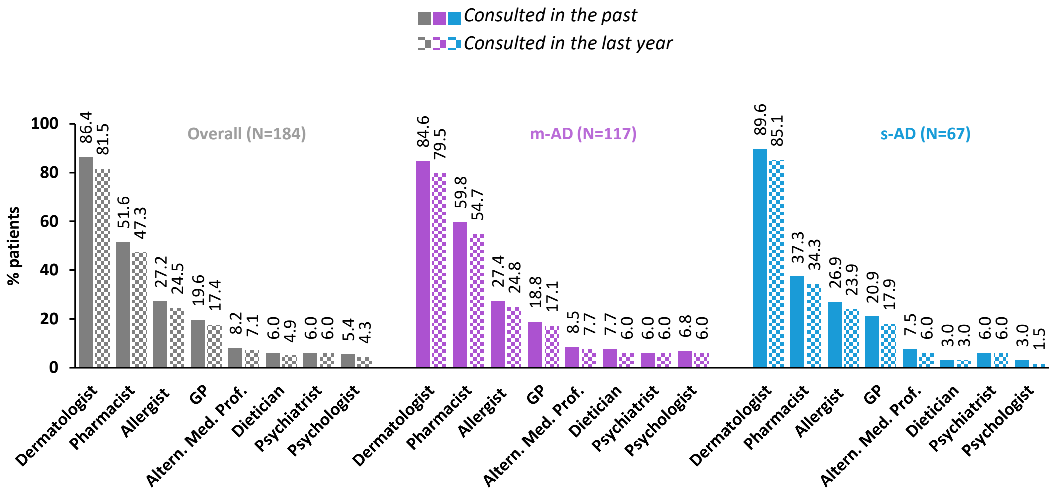

Supplementary Figure S3. HCP specialties consulted in the past, in the overall M2S-AD population and by EASI-based AD severity.

HCP specialties reported in >5% of the patients are presented, excluding pediatricians. Numbers on top of bars indicate percentage (%).

AD, atopic dermatitis; Altern. Med. Prof., professional on alternative medicine; EASI, eczema area and severity index; GP, general practitioner; HCP, healthcare professional; M2S, moderate-to-severe; m-AD, moderate AD; N, number of patients with available data; s-AD, severe/very severe AD.
